# Adolescents' perceptions of the aesthetic impact of dental fluorosis vs. other dental conditions in areas with and without water fluoridation

**DOI:** 10.1186/1472-6831-12-4

**Published:** 2012-02-10

**Authors:** Michael G McGrady, Roger P Ellwood, Michaela Goodwin, Nicola Boothman, Iain A Pretty

**Affiliations:** 1 School of Dentistry, University of Manchester, Manchester M13 9PL, UK; 2 Colgate Palmolive Dental Health Unit, 3A Skelton House, Lloyd Street North, Manchester M15 6SH, UK

## Abstract

**Background:**

The use of fluorides for caries prevention is well established but is linked with an increased risk of dental fluorosis, some of which may be considered to be aesthetically objectionable. Patient opinion should be considered when determining impact on aesthetics. The aim of this study was to assess participant rating of dental aesthetics (from photographic images) of 11 to 13 year olds participating in an epidemiological caries and fluorosis survey in a fluoridated and a non-fluoridated community in Northern England.

**Methods:**

Consented participants were invited to rank in order of preference (appearance) a collage of 10 computer generated images on a touch-screen laptop. The images comprised an assortment of presentations of teeth that included white teeth, a spectrum of developmental defects of enamel and dental caries. Data were captured directly and exported into SPSS for analysis.

**Results:**

Data were available for 1553 participants. In general, there were no significant differences in the rank positions between the fluoridated and non-fluoridated communities, with the exception of teeth with caries and teeth with large demarcated opacities. Very white teeth had the highest rating in both localities. Overall, there was a trend for teeth with fluorosis to be ranked more favourably in the fluoridated community; for TF 1 and TF 2 this preference was significant (p < 0.001).

**Conclusions:**

The results of this study suggest teeth that are uniformly very white have the highest preference. The rankings suggest teeth with a fluorosis score of TF 1 may not be considered aesthetically objectionable to this population and age group. The image depicting a tooth with caries and the image with large demarcated opacities were deemed to be the least favoured. Participant preference of images depicting fluorosis falls with increasing severity of fluorosis.

## Background

The use of fluorides in dentistry has been associated with a decline in the prevalence of dental caries through the use of optimally fluoridated community water supplies and fluoridated oral care products. However, the presence of multiple vehicles for fluoride delivery has also been associated with concerns regarding increased prevalence of dental fluorosis in both fluoridated and non-fluoridated communities [1-4].

It has been demonstrated that exposure to fluoridated water supplies in addition to the use of fluoridated dentifrices is more effective than the use of fluoridated dentifrice alone in preventing caries [1]. However, the increase in the prevalence of enamel fluorosis has led to concerns over the risk benefit ratio with respect to the use of fluorides to reduce caries and the risk of enamel fluorosis. Studies addressing the aesthetic impact of fluorosis suggested teeth with Thylstrup and Fejerskov (TF) index scores of 3 or higher elicited concerns regarding appearance [5,6]. This was in contrast to mild fluorosis (TF index 1 or 2) [6]

In the UK, a systematic review commissioned by the government known as the York Report [7] stated the occurrence of fluorosis at water fluoride levels of 1 ppm was found to be high (predicted 48%, 95% CI 40 to 57). Of this fluorosis, the proportion considered to be aesthetically objectionable was lower (predicted 12.5%, 95% CI 7.0 to 21.5). Dental fluorosis was deemed to be perceived as a potential aesthetic problem [5] and despite the increase in prevalence of fluorosis it was not perceived by clinicians to be an important consideration, particularly for patients with less severe presentations [8]. A recent review of the literature relating to fluorosis aesthetics and Oral Health Related Quality of Life (OHRQoL) concluded very mild and mild fluorosis was not associated with negative effects on OHRQoL, but more severe presentations of fluorosis was consistently reported less favourably [3].

It is probable there are differences in perception of aesthetics between clinicians and patients [9-11], but there is inconsistency in the literature with respect to this [5]. However, this does not take into consideration the different social norms and beliefs between the various study populations that could have an impact upon the outcome of perception of aesthetics, nor does it reconcile the desire to record clinically significant or aesthetically objectionable fluorosis with the need to record all forms of fluorosis for epidemiological purposes.

Nevertheless, a report from the Medical Research Council (UK) [12] that followed the York Report added a further qualification on the viewpoint of the aesthetic component of fluorosis by stating:

"Further studies should determine the public's perception of dental fluorosis with particular attention to the distinction between acceptable and aesthetically unacceptable fluorosis."

The ability of a group of lay persons to reliably comment upon the aesthetic appearance of fluorosis is difficult to assess. The level of agreement between study groups which include lay people has been shown to reduce as the TF score (severity of fluorosis) increases [13].

Studies have highlighted the effects of facial features, viewing distance and tooth morphology and alignment as factors that can influence an individual's perception of aesthetics [14-16]. The display media employed may also have an effect on a viewer's capacity to rate images with image magnification, and ambient lighting acting as confounding factors. Whilst standardized techniques can be used to capture images, the decision to capture images of wet or dry teeth will also have an effect on the degree of hypomineralization that is recorded.

The aim of this study was to evaluate participant rating of dental aesthetics. The main focus was the rating of aesthetics relating to enamel fluorosis in sample populations residing in a fluoridated and a non-fluoridated urban communities.

## Methods

Participants were males and females aged 11 to 13 who were participating in an epidemiological survey of caries and fluorosis prevalence and severity in an urban population with water fluoridation (Newcastle Upon Tyne, UK) and without (Greater Manchester, UK). The study took place between March 2008 and December 2009. Ethical approval was obtained from the University of Manchester Committee on the Ethics of Research on Human Beings (ref: 07952) to include participant assessment of fluorosis aesthetics as part of the survey outcome. Written informed consent was obtained from the participants following an opportunity for parents to object to their child's participation via a postal return of pre-prepared slips. This approach to consent was deemed to be acceptable owing to the non-interventional nature of the study and participant capacity could be clearly demonstrated. The informed consent processes were commensurate with those used for a national surveillance programme (NHS Dental Epidemiological Programme for England) conducted in the same age group.

### Screening and selection of participants

In order to obtain balance between the two cities with respect to social deprivation, schools were identified based upon the percentage Free School Meals Entitlement (%FSME). The %FSME data was obtained through the schools and Local Authorities and has been used as a variable for estimating social deprivation in resource allocation for schools in Northern Ireland [17]. During recruitment the participants provided postcode details that were used to obtain Index of Multiple Deprivation (IMD) scores. Eligible participants were required to be lifelong residents in their geographical location (self reported).

### Aesthetics perception assessment

**Figure 1:**
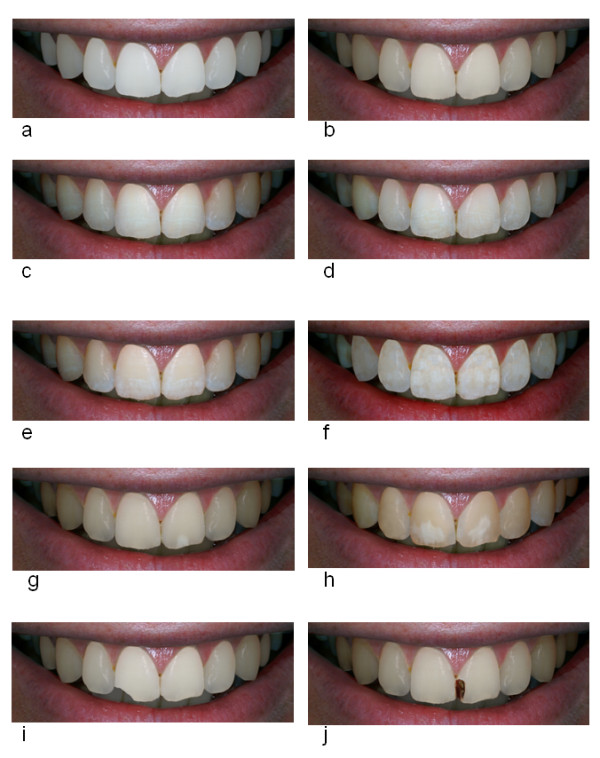
**Images selected for study**. Note how the images share a common base image with computer generated conditions stencilled over. 1a: very white teeth; 1b: teeth shade A1; 1c teeth with fluorosis TF1; 1 d: teeth with fluorosis TF2; 1e: teeth with fluorosis TF3; 1f: teeth with fluorosis TF4; 1 g: medium sized demarcated opacity on one tooth; 1 h: large demarcated opacities on both central incisors; 1i: teeth shade A1 with a chip on incisal edge of one tooth; 1j: teeth with carious lesion on one tooth.

Recruited participants were asked to complete a brief computer based assessment of tooth aesthetics. The assessment comprised of a montage of ten "life size" images of teeth with a variety of dental conditions which the participants were asked to rate in order of preference with respect to appearance i.e. which they preferred the appearance of ranked or rated best (Figure 1). The images were computer simulated images with "stencils" of dental conditions overlaid onto a base image of an individual's teeth. This ensured the size and contour of the teeth as well as the lips and gingival tissues were consistent across the images. Every participant used the same computer to ensure the image size and the viewing distances were consistent for each participant i.e. a viewing screen dimension of 12.1 inches with a 12 to 18 inches viewing distance of images which were 1750 × 1000 pixels in dimension to represent life size images. The ten images are illustrated and described in Figure 1.

The images were loaded into a programme written in Microsoft Visual Studio (Microsoft Corp, Seattle, USA) running on an IBM ThinkPad (Lenovo X60). Each participant was invited to enter their unique participant identifier into the computer which then displayed the ten images in a randomized order on the screen. The participants were asked to independently rate the images in order of preference by dragging a number between 1 and 10 to the images using a touch screen pen. The participants were free to alter their preferences by relocating the numbers between the images. Once the participants were satisfied with their selection they were asked to save their preferences which downloaded the information to a database and exited the programme in readiness for the next participant. Time constraints and school curriculum congestion prevented an assessment of reliability.

### Data management and analysis

The database was exported to SPSS for analysis. The mean ranks were calculated for each of the images and analysis performed to explore patterns in the data with respect to fluoridation status, deprivation and gender by performing t-tests between data generated for participants in the two localities. A series of non-parametric pairwise comparisons were performed to explore image preferences by the numbers of participants who rated a nominated image higher than the remaining images.

## Results

**Figure 2:**
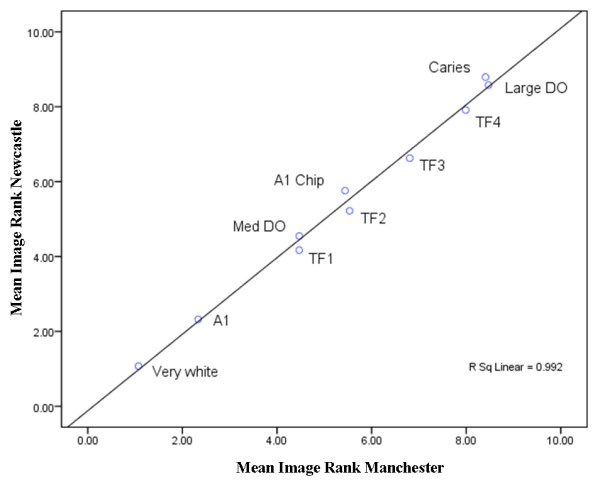
**Mean rank for each image for both cities demonstrating level of agreement of participants between cities suggesting participants in Newcastle are more tolerant of milder presentations of fluorosis compared to Manchester**. (DO = Demarcated Opacity; A1 Chip = chip on incisal edge).

In total 1646 participants from 15 schools (10 Newcastle; 8 Manchester) from the main survey were available to participate and consented to complete the aesthetics assessment (100% response rate). After data cleaning data for 1553 participants were available for analysis. The reason for lost data was predominantly owing to incorrectly entered subject numbers or clerical errors preventing the study team linking data to participants. Demographics for the participants are described in Table 1. Descriptive statistical analysis provided mean image ranks for Newcastle (fluoridated), Manchester (non-fluoridated) and for all participants and are displayed in Table 2. Overall, participants expressed the highest preference for very white teeth and teeth Vita shade A1. Participant rating of the images of teeth with caries or large demarcated opacities demonstrated the worst rating. Teeth with a fluorosis severity of TF 1 had an overall rank position of third. However, there was no clear pattern of preference amongst the remaining images with clustering of mean ranks and greater variability. In general, there were no differences in the rank positions between the two cities, with the exception of the rank positions of teeth with caries (Figure 1j) and teeth with large demarcated opacities (Figure 1h) which were ranked 9 and 10 in Newcastle but in Manchester caries and large demarcated opacities were ranked 10 and 9 respectively. Similarly, the rankings of teeth with a chipped incisal edge (Figure 1i) and teeth with fluorosis score TF 2 (Figure 1d) are reversed between the two cities. Comparison of the mean ranks for each image between the two cities revealed significant differences for images of teeth with fluorosis severities TF1 and TF2 (Figure 1c, and 1d respectively). There were also significant differences between the cities for images of teeth with caries and teeth with a chip on the incisal edge. This is also displayed in Table 2. It should be stated where there were statistically different differences found the magnitude of the differences was relatively small. A scatter plot of the mean image ranks for the two cities is illustrated in Figure 2. The scatter plot illustrates the differences in mean image ranks: images of teeth with fluorosis have a lower mean rank position in fluoridated Newcastle when compared to non-fluoridated Manchester. It may be possible to suggest fluorosis might be considered more aesthetically acceptable in fluoridated Newcastle. The image with caries was rated lower by participants in Newcastle compared to participants in Manchester.

**Table 1:** Participant demographics

City	Total Participants	Males	Females	Mean Age Years (SD)
**Newcastle**	741	367	374	12.66 (0.44)

**Manchester**	812	471*	341*	12.33 (0.65)

	1553	838	715	

* Gender imbalance in Manchester owing to the inclusion of a single sex (boys only) school

**Table 2:** Descriptive analysis: for all participants, by city and for the lowest and highest quintiles of deprivation

	Image letter (Figure 1)	City: mean rankings				
		
		Newcastle (N = 741) Fluoridated	Manchester (N = 812) Non-fluoridated	Total (1553)	Independent Samples t-test (between cities)	
					
					P value	95% CI
		**Mean (S.D.)**	**Mean (S.D.)**	**Mean (S.D.)**		

**Very White Teeth**	**a**	1.07 (0.452)	1.07 (0.536)	1.07 (0.497)	0.948	(-0.048, 0.051)

**Vita shade A1**	**b**	2.32 (0.945)	2.34 (1.031)	2.33 (0.991)	0.678	(-0.120, 0.078)

**Fluorosis TF1**	**c**	4.17 (1.529)	4.47 (1.618)	4.33 (1.583)	< 0.001*	(- 0.451, - 0.137)

**Medium demarcated opacity**	**g**	4.55 (1.547)	4.48 (1.665)	4.51 (1.61)	0.406	(-0.092, 0.228)

**Fluorosis TF2**	**d**	5.22 (1.620)	5.54 (1.678)	5.39 (1.658)	< 0.001*	(- 0.485, - 0.156)

**Vita A1 chipped incisal edge**	**i**	5.75 (2.28)	5.43 (2.285)	5.59 (2.287)	< 0.005*	(0.01, 0.554)

**Fluorosis TF3**	**e**	6.63 (1.512)	6.81 (1.504)	6.72 (1.51)	0.018	(-0.332, -0.032)

**Fluorosis TF4**	**f**	7.92 (1.453)	7.99 (1.639)	7.95 (1.553)	0.374	(-0.225, 0.085)

**Large demarcated opacity**	**h**	8.58 (1.395)	8.47 (1.523)	8.52 (1.464)	0.132	(-0.034, 0.258)

**Teeth with Caries**	**j**	8.79 (1.614)	8.41 (1.901)	8.59 (1.78)	< 0.001*	( 0.203, 0.556)

	Image letter (Fig 1)	**Deprivation: mean rankings**				
		
		Lowest Quartile Deprivation (n = 308)	Highest Quartile Deprivation (n = 325)	Independent Samples t-test (between deprivation quartiles)		

		Mean (S.D.)	Mean (S.D.)	P value	95% CI	

**Very White Teeth**	**a**	1.10 (0.689)	1.09 (0.612)	0.931	(-0.081, 0.089)	

**Vita shade A1**	**b**	2.38 (1.089)	2.40 (1.006)	0.403	(-0.210, 0.085)	

**Fluorosis TF1**	**c**	4.23 (1.556)	4.37 (1.640)	0.379	(-0.334, 0.127)	

**Medium demarcated opacity**	**g**	4.21 (1.514)	4.62 (1.705)	0.001*	(- 0.633, - 0.158)	

**Fluorosis TF2**	**d**	5.55 (1.557)	5.36 (1.733)	0.131	(-0.55, 0.419)	

**Vita A1 chipped incisal edge**	**i**	5.92 (2.365)	5.30 (2.223)	0.001*	(0.253, 0.969)	

**Fluorosis TF3**	**e**	6.60 (1.497)	6.77 (1.511)	0.154	(0.369, 0.058)	

**Fluorosis TF4**	**f**	8.02 (1.486)	7.99 (1.476)	0.191	(-0.069, 0.348)	

**Large demarcated opacity**	**h**	8.38 (1.513)	8.63 (1.484)	0.072	(-0.405, 0.017)	

**Teeth with Caries**	**j**	8.61 (1.836)	8.46 (1.855)	0.447	(-0.158, 0.359)	

Rankings for images based from 1 to 10, with 1 rated best.

**Figure 3:**
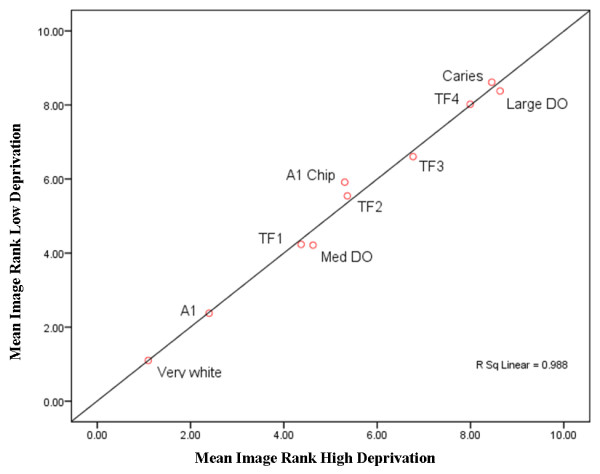
**Mean rank for each image (all participants) for the lowest and highest quartiles of deprivation**. (DO = Demarcated Opacity; A1 Chip = chip on incisal edge).

To explore the association of deprivation on aesthetics perception, the mean image ranks for all participants in the lowest and highest quartiles of deprivation (as determined by Index of Material Deprivation) were compared and shown in Table 2. After adjustment for multiple comparisons (Bonferroni), significant differences for teeth with medium demarcated opacities (p = 0.001) and teeth with a chip on the incisal edge (p = 0.001) were found between participants from the lowest and highest quartiles of deprivation. A scatter plot of the mean ranks for the images comparing the lowest and highest quintiles of deprivation is illustrated in Figure 3. The data suggests teeth with a medium demarcated opacity may be ranked less favourably by participants who are more deprived and teeth with a chip on the incisal edge are ranked more favourably by less deprived participants.

There were no significant differences in mean image ranks when looking at data for gender in this population.

A binomial analysis was carried out exploring pair-wise comparisons between each of the images to determine which image in each pair was preferred over the other. Selected data from this analysis are displayed in Table 3: for very white teeth and for teeth with a fluorosis score of TF 1 vs. each of the other images. These selections were made based upon the clear preference for very white teeth and the relative importance of assessing the impact of mild fluorosis (TF 1). The data clearly illustrates participants significantly preferred very white teeth compared to all of the other images. When exploring the data for teeth with a fluorosis score of TF 1, participants did not prefer TF 1 to tooth shade A1 or very white teeth. A majority of participants preferred TF 1 to teeth with a medium sized demarcated opacity but this preference was not statistically significant (p = 0.171). Teeth with a fluorosis score of TF 1 were significantly preferred over all remaining images.

**Table 3:** Selected binomial pairwise comparisons: depicting image preference for very white teeth and teeth with fluorosis severity TF1 against each image

	First group	Second group	Asymp. Sig. (2-tailed)
Very white vs A1	1494	59	< 0.001

Very white vs TF1	1537	16	< 0.001

Very white vs Medium DO	1544	9	< 0.001

Very white vs TF2	1545	8	< 0.001

Very white vs A1 chip	1549	4	< 0.001

Very white vs TF3	1547	6	< 0.001

Very white vs TF4	1549	4	< 0.001

Very white vs Large DO	1549	4	< 0.001

Very white vs caries	1549	4	< 0.001

	First group	Second group	Asymp. Sig. (2-tailed)

TF1 vs Very white	16	1537	< 0.001

TF1 vs A1	182	1371	< 0.001

TF1 vs Medium DO	804	749	= 0.171

TF1 vs TF2	1119	434	< 0.001

TF1 vs A1 chip	966	587	< 0.001

TF1 vs TF3	1359	194	< 0.001

TF1 vs TF4	1459	94	< 0.001

TF1 vs Large DO	1484	69	< 0.001

TF1 vs caries	1420	133	< 0.001

The columns in Table 3 represent numbers of participants who ranked the image for very white teeth or the image of TF 1 (First group) higher than the remaining images (Second group) e.g. only 59 participants ranked the image with A1 teeth higher than the image with very white teeth

## Discussion

The results of this study suggest teeth that are either very white or uniformly white have the highest rating. The ranking of images of teeth with a fluorosis score of TF 1 may lead to the inference this sample of 11 to 13 year olds do not consider milder presentations of fluorosis to be aesthetically objectionable. The very white teeth represented an unnatural presentation that could only be achieved by cosmetic procedures. Unsurprisingly, the images depicting a tooth with caries or large demarcated opacities were deemed to be the least favoured. This is consistent with previous work related to dental aesthetics [18,19] whereby teeth with mild forms of fluorosis (TF 1, TF2) were rated similarly; and such presentations of mild fluorosis were rated higher than teeth with demarcated opacities, interdental "black triangles" (related to prosthetic treatment) or caries. The remaining images provided an equivocal representation of participant preference. This is not an unusual finding with ranked data where there is a clear separation at extreme ends of the scale for the most and least preferred images and where there remains a central group of images that participants have no strong preference of one image over another. The finding that the image with a tooth with a chip on the incisal edge was ranked higher by participants who are less deprived is of interest. However, it is difficult to provide a satisfactory explanation for this phenomenon as additional contextual information was not available. For example, it was not known if a participant's decision was influenced by factors such as the effect routine restorative treatment would have on the appearance of the teeth. Consequently this image was associated with the largest standard deviation of mean rank position i.e. the most uncertainty and variation. It is important to recognize the outcome of this study was to explore participant rating or ranking of images, not to establish a level of aesthetically objectionable fluorosis. However, when exploring comparisons between the fluoridated and non-fluoridated communities it is clear when location (fluoridation status) is a factor, the participants have more difficulty rating images with fluorosis severities of TF 1 and TF 2 in terms of preference when they are compared to TF 3. This might suggest when fluorosis severity reaches threshold of TF 3 participants more reliably express a lower preference.

It should, however, be stated there are several limitations with the study design and there are issues to be raised from the interpretation of the data. The nature of the study assessment, a brief computer-based questionnaire, is not a novel technique and has been used successfully and reported elsewhere in the literature [14-16]. However the outcomes of the current study were limited to simple ranking data, associated with limitations and difficulties in analysis and interpretation as the numeric output has more limited value in analytical terms. Additional work may be undertaken to examine the use of "ties" between rating and Likert scales--although these approaches also have their limitations.

The participants who participated in the survey were self-reported lifetime residents of their locality. Therefore this analysis does not take into consideration the aesthetic perceptions of individuals who moved into a particular location. These data suggest participants who were lifetime residents in a fluoridated region may tolerate or perceive mild levels of fluorosis more favourably than individuals residing in a non-fluoridated area. Is this a phenomenon resulting from social norms and would an individual who moves from a non-fluoridated region into a fluoridated region hold the same views? Similarly, this study has not taken into account possible effects of participant ethnicity on aesthetic perception. Both of these should be considered for future work--perhaps concentrating on smaller participant numbers and a more qualitative approach.

Whilst the objective of this study was to investigate participant rating of tooth aesthetics, particularly fluorosis, it is important to make a distinction between fluorosis prevalence and severity as determined by a dental professional and what is considered to be fluorosis of aesthetic concern from the perspective of a patient. The latter is an important factor in fully determining the impact of the risk benefit ratio of an intervention such as water fluoridation or the use of fluoridated oral care products. However, it is necessary to consider all presentations of fluorosis from an epidemiological standpoint particularly when identifying trends or changes in fluorosis prevalence and severity. The choice of index employed during the assessment of fluorosis has a bearing on the determination of the prevalence and severity of fluorosis. An index which requires the drying of teeth prior to scoring such as the TF Index will result in the dehydration of hypomineralized enamel and a change in refractive index. Hence minor fluorotic opacities may not be visible when teeth are viewed wet. As a result of this phenomenon the results of this study represent an artificial scenario whereby participants are being asked to rate preference of teeth viewed as if they had been dried. It would be interesting to note any changes to participant perception if the teeth had been viewed as they would appear wetted by saliva.

In order to control the experimental environment, measures were taken to remove confounding factors. The use of a standardized base image removed the effects of tooth morphology and surrounding facial features that could impact on aesthetic perception. However, this resulted in the participants being asked to rate only a single presentation of each type of condition i.e. one image for each level of fluorosis severity, one image of caries, etc. It stands to reason different presentations of conditions could be rated differently within their classification (e.g. differing presentations of TF 2) or between images of fluorosis and different classifications such as caries or demarcated opacities. The participants also viewed images at a life size level of magnification and this was consistent throughout the study. It has already been shown in the literature that both the image magnification, the image viewing distance and the presence of other facial features all have impacts on aesthetic perception [16]. The lack of repeat assessments for reliability is a limitation of the study design. Repeat measures may have identified individuals providing spurious data e.g. the ranking of the image of a carious tooth highest. However, logistical constraints during the study prevented the assessment of reliability. The age group represented in this study occupy a significant stage of development in relation to changes in awareness of appearance and as such it is possible the age of the participant could impact upon the outcome of the study. There was no assessment of this during the study as there was a lack of contextual data such as academic ability to perform a meaningful analysis.

This study aimed to seek opinion in relation to dental aesthetics from adolescents at key stages of development for the dentition and awareness of appearance. The data would suggest gender did not significantly influence aesthetic rating in this scenario whereas deprivation status or geographical location (residing in either a fluoridated area or a non-fluoridated area) appeared to have a greater influence on aesthetic rating. Further work is needed to evaluate the relationship between the participants' rating of dental aesthetics and the influence of their own dental presentation on their aesthetic judgment. This should also incorporate the implications of fluorosis on the individual relating to dental treatment and any impact on quality of life.

## Conclusions

It is clear from the results of this study that participants have a preference for white, blemish free teeth, even within this age group many of whom are still in the mixed dentition stage. One inference from the data is the mildest presentations of fluorosis (TF 1) may not be associated with aesthetic concerns. As fluorosis severity increases (TF 2 or greater), the rating of images (and perhaps the level of acceptance) declines which is in agreement with earlier work [6,16,19-21]. However, it is not possible from the outcome of this study to determine a cut off level of fluorosis severity that is considered to be an aesthetic problem.

## Abbreviations

%FSME: Percentage free school meal entitlement; CI: Confidence interval; IMD: Index of multiple deprivation; NHS: National health service; OHRQoL: Oral health related quality of life; SPSS: Statistical package for social sciences; TF: Thylstrup & Fejerskov Index

## Competing interests

None of the authors are aware of any competing interests in the production of this manuscript.

## Authors' contributions

MGM prepared the protocol, conducted the fieldwork, was involved in the analysis of data and wrote the manuscript. RPE provided input into the study design and the manuscript. NB co-ordinated the study, conducted fieldwork and participant instruction on questionnaire completion. MG conducted the statistical analysis. IAP provided input into study design and manuscript. All authors read and approved the final manuscript.

## Pre-publication history

The pre-publication history for this paper can be accessed here:

http://www.biomedcentral.com/1472-6831/12/4/prepub
